# Varied application of intercostal trans-diaphragmatic ports for laparoscopic hepatectomy

**DOI:** 10.1371/journal.pone.0234919

**Published:** 2020-06-19

**Authors:** Hiromitsu Hayashi, Yo-ichi Yamashita, Hirohisa Okabe, Katsunori Imai, Takaaki Higashi, Kensuke Yamamura, Akira Chikamoto, Toru Beppu, Hiroshi Takamori, Hideo Baba

**Affiliations:** 1 Department of Gastroenterological Surgery, Kumamoto University, Kumamoto, Japan; 2 Department of Surgery, Yamaga City Hospital, Kumamoto, Japan; 3 Department of Surgery, Saiseikai Kumamoto Hospital, Imperial Gift Foundation, Kumamoto, Japan; Cleveland Clinic, UNITED STATES

## Abstract

**Background:**

The ribcage and diaphragm are mechanical barriers for laparoscopic access during hepatectomy. Here, we introduce the varied application of intercostal trans-diaphragmatic ports during laparoscopic hepatectomy, and describe the management of intercostal ports with key technical points.

**Methods:**

From January 2013 to December 2017, 180 patients underwent laparoscopic hepatectomy. In 32 of these patients (17.8%), intercostal ports (31 right and one left) were applied, and we analyzed the feasibility and safety of intercostal ports during laparoscopic hepatectomy.

**Results:**

The main tumor location was segment VII and VIII (78%). The major type of laparoscopic hepatectomy was partial hepatectomy (91%). In the majority of cases (66%) the number and size of intercostal trocars was a single 5-mm port. The median operative time and blood loss were 232 min and 50 mL, respectively. A chest drain was placed via the hole of the intercostal port on the chest wall in two cases (6.3%). The median duration of the post-operative hospital stay was 6 days. There was no conversion, and a pure laparoscopic hepatectomy was achieved in all cases. There was no mortality. As for complications due to the application of intercostal ports, an asymptomatic pneumothorax was detected in only one case, and it was cured by conservative treatment.

**Conclusions:**

The ribcage and diaphragm could be overcome as barriers to laparoscopic access by the placement of intercostal ports with minimal access during laparoscopic hepatectomy. The use of an intercostal port and proper management allows for a feasible approach and safe resection during laparoscopic hepatectomy.

## Introduction

Laparoscopic hepatectomy for liver tumors may yield similar oncologic outcomes to conventional open hepatectomy, while having advantages such as lower blood loss, fewer post-operative complications and shorter hospital stay[1]. On the other hand, the surgical difficulty of each laparoscopic hepatectomy depends upon a variety of factors, ranging from the style and extent of liver resection to the tumor condition (size, location and proximity to major vessels) and the background liver condition (liver functional reserve, fibrosis, steatosis, deformity and adhesion after previous resection). A novel difficulty score for laparoscopic hepatectomy has been proposed and is calculated by summating applicable scores for tumor location, the extent of liver resection, liver function, tumor size and tumor proximity to major vessels[2]. In this scoring system, a tumor located in a postsuperior position such as segment VII and VIII earns a higher score than an anterolateral tumor, due to the difficulties presented by its location[2]. These operative findings are due to the mechanical barriers of the ribcage and diaphragm for laparoscopic access to a postsuperior lesion. Additionally, repeated hepatectomy has also been difficult in laparoscopic approach because of deformity and adhesion following a previous resection.

To overcome the mechanical barriers presented by the ribcage and diaphragm, the placement of ports through the intercostal spaces is a minimally invasivene option. With a direct view of the lesion, additional ports through intercostal spaces allow the placement of instruments along all transection planes.

Here, we report on a series of 32 patients in whom the use of intercostal trans-diaphragmatic ports allowed for a safe approach in a variety of laparoscopic hepatectomies with minimal access. We describe the feasibility and safety with key technical points.

## Methods

### Patients

From January 2013 to December 2017, 180 patients underwent laparoscopic hepatectomy at Kumamoto University and Saiseikai Kumamoto Hospital. In 32 (17.8%) of them, intercostal trans-diaphragmatic ports were applied during laparoscopic hepatectomy, and we retrospectively analyzed the feasibility and safety of such ports during surgery. This retrospective cohort study was approved by the Kumamoto University Hospital Ethics Committee (approved number is #1789). All data were fully anonymized before we analyzed them. **Table 1** presents the baseline patient and clinical characteristics of the 32 patients. Past history of a laparotomy was detectable in 25 cases (78%). In 15 cases (47%), upper abdominal surgery was performed. Seven cases (22%) had a past history of hepatectomy.

**Table 1:** Background characteristics of patients with intercostal trocars in laparoscopic hepatectomy (N = 32).

Age (years)	71 (54–87)
Gender (male / female)	27 / 5
Past laparotomy	25 (78%)
Past upper laparotomy	15 (47%)
Past hepatectomy	7 (22%)
Past open hepatectomy	5 (16%)
Past laparoscopic hepatectomy	2 (6%)
Type of liver tumor (HCC / CLM / others)	20 / 9 / 3
Targeted tumor size (mm)	20 (6–60)
Targeted tumor number	1 (1–5)
Targeted main tumor location (S2/5/6/7/8)	1 / 2 / 4 / 13 / 12
Procedure	
Partial hepatectomy (single)	26 (82%)
Partial hepatectomy (double)	2 (6%)
Posterior sectorectomy	3 (9%)
Partial hepatectomy + left lateral segmentectomy	1 (3%)
Available Pringle method	20 (63%)
Additional procedure	
Radio frequency ablation	1
Laparoscopic colorectomy	2
Laparoscopic cholecystectomy	2
Abdominal wall hernia repair	1
Intercostal port	
Single 5-mm port	21 (66%)
Double 5-mm ports	3 (9%)
Single 12-mm port	3 (9%)
5-mm and 12-mm ports	4 (13%)
Double 12-mm ports	1 (3%)

Values are expressed as median (range) or case number (percent).

HCC, hepatocellular carcinoma; CLM, colorectal liver metastasis

### Operative technique

#### Port setting including intercostal transthoracic port

**Fig 1:**
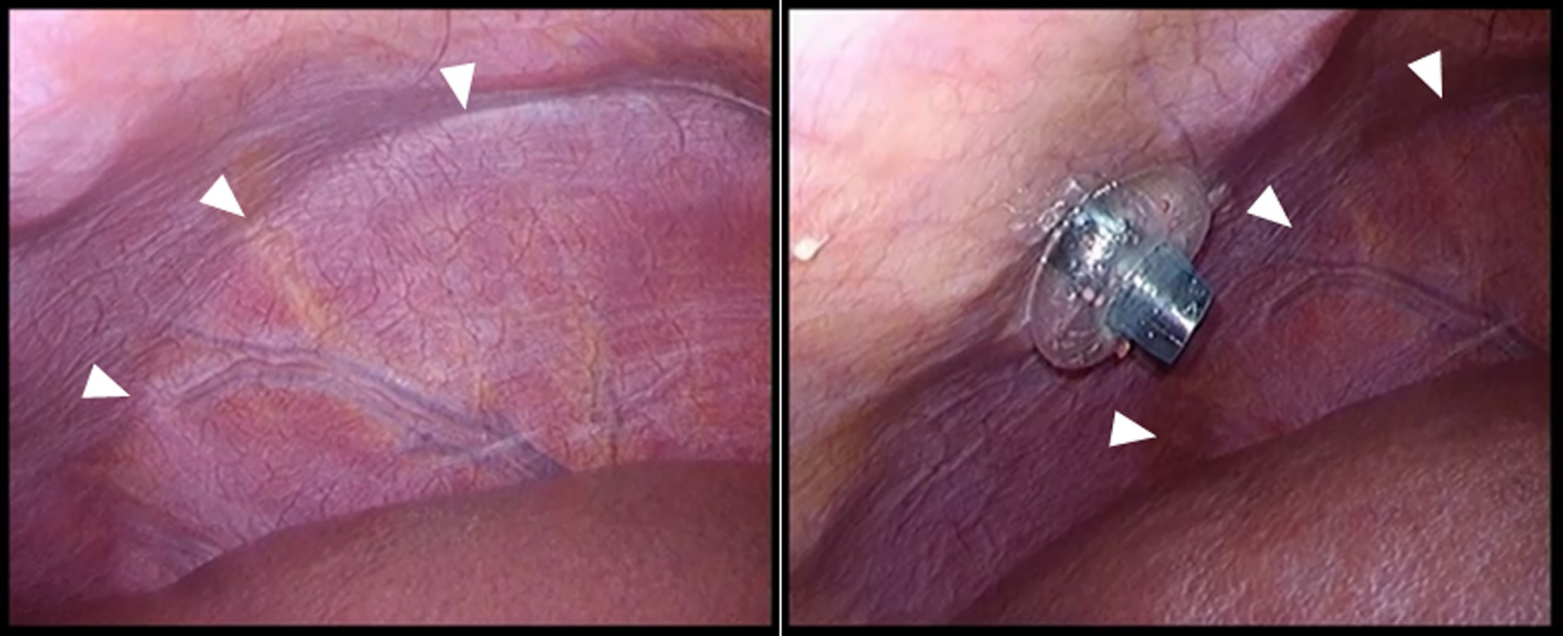
Lung edge can be confirmed by ventilation. Arrowheads indicate the lung edge.

Initial access was obtained by standard abdominal laparoscopic techniques in order to visually assess the liver and its surroundings. Five ports were routinely used for laparoscopic hepatectomy. If necessary, an additional port was introduced during surgery. One or two 5-mm or 12-mm trocars were used as the intercostal transthoracic port to allow the ingress of instruments to further assess the dome of the liver and resection line. Of the five ports, the intercostal transthoracic port was usually placed last. Prior to inserting the intercostal port, laparoscopic ultrasound was routinely used to define the size and location of hepatic tumors and their proximity to major vascular structures, and to ensure that an adequate surgical margin could be obtained. If necessary, the liver was mobilized as minimally required prior to insertion of the intercostal port. For the intercostal transthoracic port, a 5-mm or 12-mm balloon port (Covidien, Mansfield, MA, USA) was used, as the balloon can be inflated and ports retracted, pulling the diaphragm back against the chest wall and enlarging the field of view. Several deep lung ventilations were introduced by an anesthetist to confirm the edge of the lung and to determine the location of the intercostal port setting, as exemplified in **Fig 1**. An external echo examination could also be applied to confirm the edge of the lung. During an exhalation, the intercostal transthoracic port was then carefully placed intraperitoneally through the diaphragm.

#### Parenchymal transection during laparoscopic hepatectomy

The Pringle maneuver was routinely used to minimize blood loss during hepatic surgery by clamping the vascular pedicle as tightly as possible. The line of transection was marked with electrocautery and parenchymal transection performed with a laparoscopic Cavitron ultrasonic surgical aspirator (CUSA) (Valleylab, Boulder, CO, USA) or by the crushing method using a BiClamp (ERBE, Tubingen, Germany). An articulating sealing device (Enseal; G2, Ethicon Endo-Surgery, Cincinnati, OH, USA) was also used during laparoscopic hepatectomy. The surgical margin of the resection was confirmed with laparoscopic ultrasound during hepatectomy. Hem-o-Lok clips (Weck Surgical Instruments, Teleflex Medical, Durham, NC, USA) were applied to larger vessels. The specimen was placed in a retrieval bag and extracted through a pre-existing scar or an extension of a port site wound. An intrabdominal drain was routinely placed. Prophylactic systemic antibiotic therapy with 1 g cefmetazole was administered routinely immediately before surgery and twice daily for the first two post-operative days.

#### Removal of the intercostal transthoracic port, suturing of the diaphragmatic port site and chest drain placement

After the pneumoperitoneum was stopped, the intercostal trans-diaphragmatic port was finally removed under direct vision. The 5-mm diaphragmatic port site was not routinely closed if the site was covered by the remnant liver. On the other hand, in the case of a 12-mm intercostal port, closure of the diaphragmatic port site was laparoscopically performed with an absorbable suture. A chest drain was not routinely placed unless air infiltrated the thoracic cavity after removal of the intercostal trans-diaphragmatic port. In such a case, a chest drain was placed via the hole of the port site on the chest wall, and removed on the day after laparoscopic hepatectomy.

#### Definition of surgical outcomes

Surgical outcomes included blood loss, operative time, post-operative hospital stay, morbidity, and mortality in laparoscopic hepatectomy. Severe complications were defined as Grade III or greater according to the Clavien–Dindo classification[3]. Mortality referred to any death occurring during the same hospital stay.

### Statistical analysis

Statistical analysis was performed using SPSS Version 25.0 (SPSS, Chicago, IL, USA). Continuous variables were expressed as the median (range) and compared using the Mann–Whitney U-test. Categorical variables were analyzed using the chi-squared or Fisher's exact test as indicated. A *P*-value of < 0.05 was considered to indicate statistical significance.

## Results

### Varied application of intercostal diaphragmatic port according to the tumor location

**Fig 2:**
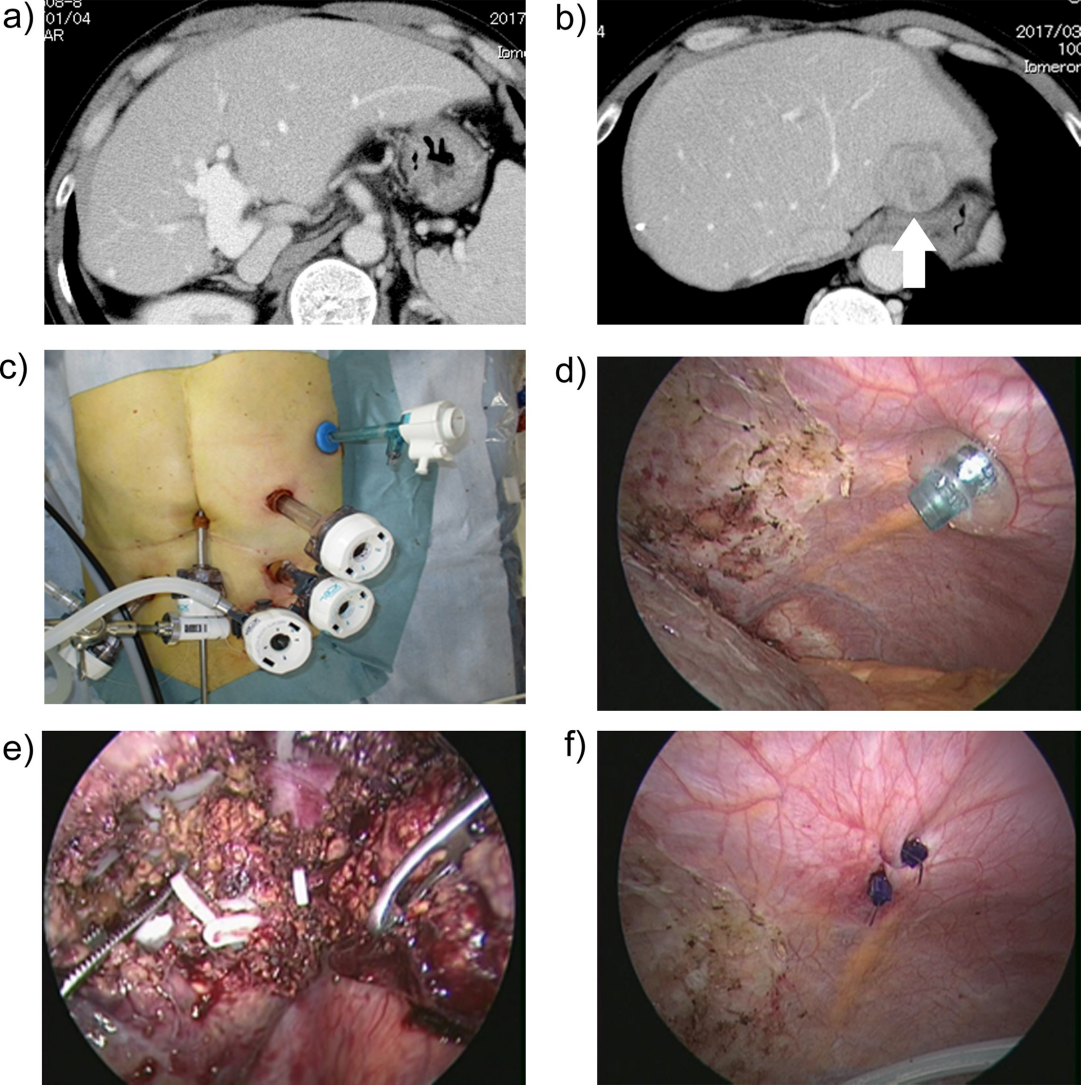
Application of a left intercostal diaphragmatic port in repeated hepatectomy. a), b) Enhanced computed tomography shows a recurrent hepatocellular carcinoma located in segment II after open right hemi-hepatectomy. An arrow indicates the recurrent tumor. c) Extracorporeal view of the left intercostal port.d) Intracorporeal view of the left intercostal port.e) View during repeated hepatectomy. f) Suture of the port hole.

**Fig 3:**
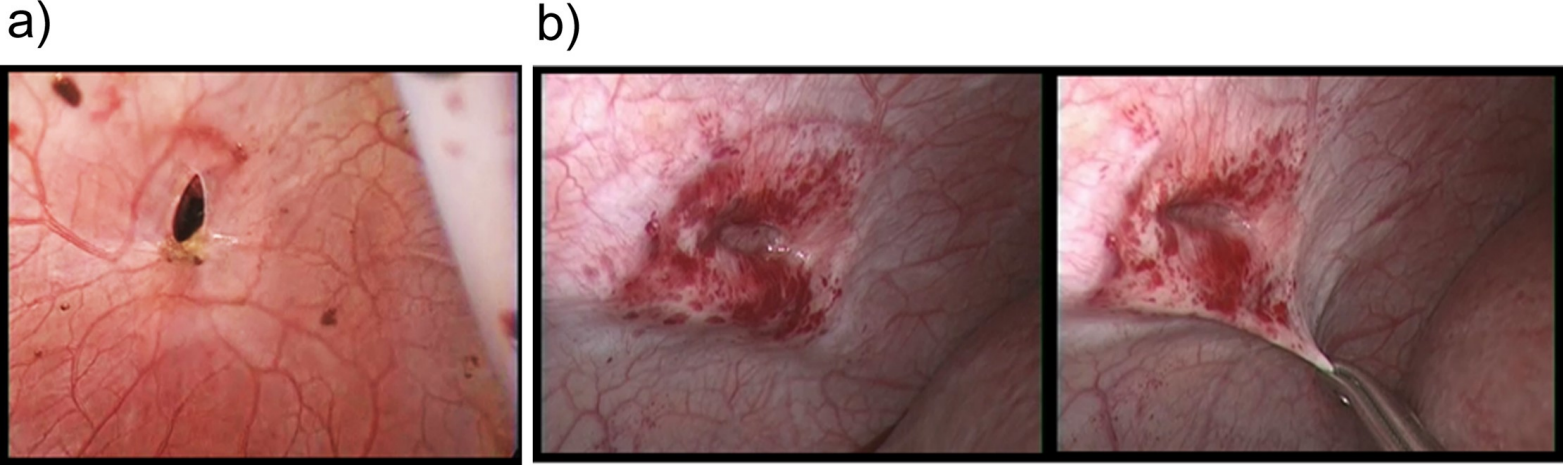
Natural course of a non-sutured 5-mm intercostal port site. a) Immediately after removal of an intercostal port. b) Six months later.

The tumor location was segment II (one case), V (two), VI (four), VII (13) and VIII (12) (**Table 1**). Thus, in cases with intercostal ports, the main tumor location was segment VII and VIII (25 cases, 78%). Of the 32 patients, right intercostal trocars were used in 31 patients (97%). In one case, a left intercostal trocar (5 mm) was introduced for recurrent hepatocellular carcinoma in segment II after open right hemi-hepatectomy (**Fig 2**). In this case with a left intercostal trocar, the diaphragmatic port site was laparoscopically closed with an absorbable suture because the site was not covered by the remnant liver. The major type of laparoscopic hepatectomy was partial hepatectomy (91%) (**Table 1**). The remaining three cases (9%) underwent a laparoscopic posterior sectorectomy. The number and size of intercostal trocars was a single 5-mm port in the majority of cases (66%). Two intercostal trocars (double 5-mm ports, 5- and 12-mm ports, and double 12-mm ports) were also used in seven cases (22%) (3 cases, 3 cases, and 1 case, respectively). Recently, a 12-mm port was not used for an intercostal trocar if possible. Coincidentally, we experienced the natural course of a non-sutured intercostal diaphragmatic trocar port (5 mm) at the time of 2nd laparoscopic hepatectomy for a recurrent tumor. In that case, the port hole was completely covered by a peritoneum at six months after the initial laparoscopic hepatectomy with an intercostal port (**Fig 3**).

### Surgical outcomes and a complication due to the application of intercostal ports

For all 32 cases, the median operative time was 232 min (range, 68–400 min) and the blood loss was 50 mL (5–1300 mL) (**Table 2**). The median duration of the post-operative hospital stay was 6 days (range, 4–17 days). A severe post-operative complication occurred in one case (post-operative bleeding). In this case, laparoscopic hemostasis was performed (Grade IIIB in Clavien–Dindo classification). There was no mortality. There was no conversion, and a pure laparoscopic hepatectomy was achieved in all cases. As for complications due to application of intercostal ports, there were no occurrences of lung injury or clinical pneumothorax, and no significant pleural effusion. In only one case, an asymptomatic and minor pneumothorax was detected by a post-operative X-ray examination, and it was cured by conservative treatment. Moreover, none of the patients displayed diaphragmatic hernia during the observation period (median, 32.5 months; range, 16.4–75.4 months). Although a chest drain was not routinely placed except when air infiltrated the thoracic cavity after the removal of intercostal trans-diaphragmatic ports, a chest drain (12 Fr) was placed via the hole of the intercostal port on the chest wall in two cases (6.3%). In one case, this was due to air infiltration into the thoracic cavity after removal of the intercostal port. In the other, a prophylactic chest drain was placed for post-operative pleural effusion due to a cirrhotic liver after posterior sectorectomy.

**Table 2:** Surgical outcomes (N = 32).

Operative time (min)	232 (68–400)
Blood loss (mL)	50 (5–1300)
Blood transfusion	3 (9%)
Conversion	0
Chest drain placement	2 (6%)
Morbidity	1 (3%) (post-operative bleeding)
Intercostal port-related complication	1 (3%) (pneumothorax: Grade 1)
Mortality (%)	0
Post-hospital stay (days)	6 (4–17)

The values were expressed as median (range) or case number (percent).

Morbidity was defined as Clavien–Dindo classification ≥ Grade III.

## Discussion

The present study describes vaired application of intercostal ports, not only for liver tumors located in postsuperior areas such as segment VII and VIII using a right intercostal port, but also for a repeated hepatectomy using a left intercostal port. Indeed, the tumors in the present study were located in a variety of liver segments: segment II (3%), V or VI (19%), and VII or VIII (78%). Conventional laparoscopic hepatectomy without intercostal port is enough to manage the liver tumours not locating at Segment VII and VIII by the liver mobilization. However, the liver mobilization is occasionally difficult due to postoperative adhesion especially in a repeated hepatectomy. When the conventional approach has the difficulty in adjusting the axis of the forcep or CUSA to adequate towing direction or transection line because of the adhesion, the intercostal trans-diaphragmatic port was chosen. In all 32 patients, a pure laparoscopic hepatectomy was successfully achieved with no conversion and no mortality, although one case (3%) had a severe post-operative complication requiring laparoscopic hemostasis for post-operative bleeding from the surface of liver resection. Regarding complications associated with intercostal ports, there were no occurrences of lung injury, clinical pneumothorax or significant pleural effusion in this study. A chest drain is was not routinely placed except for one case in which air infiltrated the thoracic cavity after removal of an intercostal port, as in previous reports (**Table 3**). In one case only (3%), an asymptomatic pneumothorax was detected by a post-operative X-ray examination; however, this case was cured by conservative treatment. In our management, the intercostal port was finally removed under direct vision after the pneumoperitoneum ceased. In our experience, a delayed termination of pneumoperitoneum can be the cause of air infiltration into the thoracic cavity and a post-operative pneumothorax. In such a case, a chest drain should be placed via the hole of the port site on the chest wall, and it can be removed on the next day or later after laparoscopic hepatectomy. Indeed, such a situation occurred in one case. In this case, a chest drain was placed, and it was removed the day after operation without any complication.

**Table 3:** Application of intercostal trocars for laparoscopic hepatectomy in current and previous studies.

**Author**	**Cases**	**Thoracic drain**	**Intercostal trocar site**	**Morbidity**
Lee et al.[4]	5	Not described	Not described	None
Chiow et al.[5]	8	Not routinely inserted	Sutured	None
Ichida et al.[6]	14	Not routinely inserted	Sutured	None
Hirokawa et al.[7]	23	Not inserted	Not sutured	4%
Present study	32	Not routinely inserted	Not sutured	3%

Morbidity was defined as Clavien–Dindo classification ≥ Grade III.

Primary closure of an intercostal diaphragmatic port is controversial (**Table 3**). In this study, closure of the 12-mm diaphragmatic port was laparoscopically performed with an absorbable suture. On the other hand, the 5-mm diaphragmatic port site was not routinely closed if it was covered by the remnant liver. If a primary closure of the diaphragmatic port site can be performed easily, we recommend the primary closure of all the diaphragmatic port sites. However, it is sometimes difficult to suture the 5-mm diaphragmatic port site under the stop of pneumoperitoneum and a restricted operation field. It can be left as a non-closure in case that the diaphragmatic port site is covered by the remnant liver. Indeed, there was no severe post-operative pneumothorax due to the non-suture of a 5-mm diaphragmatic port site in our experience. The diaphragmatic hernia can be an intercostal port-related complication. In the present study, with a median observation period of 32.5 months, no diaphragmatic hernia occurred due to an intercostal port. Coincidentally, we could confirm the natural course of a non-sutured intercostal 5-mm diaphragmatic port at the time of 2nd laparoscopic hepatectomy for a recurrent tumor. In this case, the port hole was completely covered by a peritoneum at six months after the initial laparoscopic hepatectomy. We will continue the to monitor the possible occurrence of a diaphragmatic hernia due to the intercostal port in the future. Thus, the feasibility and safety of an intercostal diaphragmatic port were described in the present study. We have also provided details of the management to avoid intercostal port-related complications.

In conclusion, an intercostal trans-diaphragmatic port can be applied in a variety of situations such as postsuperior liver tumor laparoscopy and repeated hepatectomy. The ribcage and diaphragm do not act as barriers to laparoscopic access when intercostal ports with minimal access are placed during laparoscopic hepatectomy. The use of an intercostal port and proper management allow for a feasible approach and safe resection during laparoscopic hepatectomy.

## Supporting information

S1 Table Consecutive 32 cases with intercostal trocars during laparoscopic hepatectomy. (DOCX)

## Abbreviations

CUSA: Cavitron ultrasonic surgical aspirator
