# Supplementary material for: Varied application of intercostal trans-diaphragmatic ports for laparoscopic hepatectomy
Source: PLoS One. 2020 Jun 19;15(6):e0234919. doi: 10.1371/journal.pone.0234919 (PMC7304607; doi:10.1371/journal.pone.0234919)
Supplement: S1 Table — (DOCX) [file pone.0234919.s001.docx]

**Supplemental Table 1. Consecutive 32 cases with intercostal trocars during laparoscopic hepatectomy**

Age Sex Tumor location Port Procedure Size Number Pringle Time Blood loss Thoracic Tube POD Morbidity ≥CDIII

73 M HCC S5 12mm Partial+LC 29 1 NA 361 25 － 8 －

67 M HCC S7/8 12mm x 2 Partial+Hernia 25 3 + 332 200 － 9 －

81 M HCC S7/8 5mm,12mm Partial+RFA 20 4 NA 239 10 － 9 －

64 M HCC S8 12mm Partial+lap-LAR 11 1 NA 230 200 － 17 －

75 M HCC S8 5mm,12mm Partial 11 1 NA 126 5 － 8 －

64 M mCRC S8 12mm Partial 20 2 + 266 5 － 9 －

79 F HCC S8 5mm Partial 23 1 + 372 1300 － 14 Bleeding

71 M HCC S7 5mm Partial 30 1 + 288 1100 － 8 －

73 M HCC S6 5mm Partial 13 1 + 321 900 － 6 －

77 M HCC S5,S6 5mm x 2 Partial x 2 24 2 NA 317 200 － 13 －

81 M HCC S6 5mm Partial 13 1 NA 154 50 － 8 －

70 M mCRC S7 5mm Partial 18 1 + 143 50 － 7 －

77 F mCRC S8 5mm Partial 30 1 + 162 50 － 7 －

61 M HCC S7 5mm, 12mm Partial 11 1 + 234 50 － 6 －

56 M mGC S6 5mm Partial 6 1 NA 116 50 － 6 －

66 M mCRC S7,S8 5mm, 12mm Partial x 2 10 2 NA 211 385 － 6 －

75 M mCRC S7 5mm Partial+lap-HAR 15 1 NA 330 300 － 8 －

61 F Benign S7 5mm x 2 Partial 15 1 + 135 5 － 4 －

76 M mCRC S2/3,S7 5mm Partial +LLR 33 3 NA 311 235 － 4 －

87 F HCC S8/4 5mm Partial 18 1 + 214 50 － 5 －

57 M HCC S2 5mm Partial 40 1 NA 153 100 － 4 －

69 M HCC S8 5mm Partial 26 1 + 81 30 － 4 －

67 M HCC S7/8 5mm Partial 20 1 + 187 150 － 4 －

74 M Benign S6/7 5mm Posterior sector 15 5 + 337 100 － 9 －

69 M mCRC S8 5mm Partial 25 1 + 129 50 － 4 －

60 M HCC S6/7 5mm Posterior sector 35 1 + 400 680 － 6 －

85 M HCC S8 5mm Partial 12 1 + 272 50 － 4 －

54 F Benign S6/7 5mm Posterior sector 60 1 + 310 150 + 4 －

62 M HCC S8 5mm Partial 30 1 + 252 100 － 5 －

80 M HCC S8 5mm Partial 19 1 NA 68 5 － 4 －

68 M mCRC S7/1 5mm x 2 Partial 25 1 + 192 50 + 7 －

75 M HCC S8 5mm Partial 18 1 + 194 50 － 4 －
